# Wearable Real‐Time Translation in Rhinology: A Comparative Study of Accuracy and Efficiency

**DOI:** 10.1002/alr.70193

**Published:** 2026-05-28

**Authors:** Emma J. Anisman, Nickolas Pudik, Laura Palacio‐Morales, Abdulghafoor Alani, Yanmin Qu, Chang Liu, Agustina Arce, Shin Heng Teresa Chan, Benjamin Bitner, Marc Rosen, Gurston Nyquist, Elina Toskala, Mindy Rabinowitz

**Affiliations:** ^1^ Department of Otolaryngology—Head and Neck Surgery Thomas Jefferson University Hospital Philadelphia Pennsylvania USA

## 1 Background

**Key Points**

- Wearable translation devices may soon improve access to and efficiency of medical translation.
- Meta AI glasses or Apple AirPods 3 were used to translate mock encounters.
- AirPods performed well, scoring better in Spanish than Mandarin and better than Meta glasses.

Language barriers pose significant challenges to patients, resulting in limited access to care, lower healthcare utilization, and poorer health outcomes [1]. In‐person interpreters are often underutilized due to efficiency and availability concerns, prompting the use of alternative solutions [2].

In 2025, Apple AirPods and Meta AI glasses incorporated real‐time “live translation” features into widely available wearable devices. This feature allows the wearer to hear near real‐time translation of the target language rather than requiring the speaker to pause for translation. This study evaluates the quality, usability, and efficiency of two recent wearable live translation devices in a rhinology clinical setting for Spanish‐ and Chinese‐speaking populations.

## 2 Methods

Three mock encounters between English‐speaking providers and non‐English‐speaking standardized patients were designed by a team of rhinologists and clinical translators. Scripts were developed for common rhinology chief complaints: epistaxis, chronic rhinosinusitis (CRS), and chronic rhinosinusitis with nasal polyps (CRSwNP), with consent for surgery. Medical translators served as both standardized patients and physicians.

Each encounter was performed using Apple AirPods 3, Meta AI glasses, and an in‐person translator. The AirPods were tested in Spanish and Mandarin Chinese, while the Meta glasses were tested in Spanish only, as Mandarin translation was not available. All encounters were timed and graded for accuracy. The in‐person translator encounters served as the time reference standard only and were not scored.

Translations were evaluated sentence‐by‐sentence by medical interpreters using a five‐point scale (5 indicating best performance) across four categories as described in Martos et al. [3, 4]: fluency (flow of speech), adequacy (completeness and contextual appropriateness), meaning (translation fidelity), and severity (likely clinical impact of any error). Clinically significant errors were defined as error severity from 1 to 3, 1 being the worst [4]. The usability of each device was graded by the physician and patient on the System Usability Scale (SUS) [5].

Statistical analyses were performed using IBM SPSS Statistics. AirPods and Meta performance were compared in Spanish using a Wilcoxon signed‐rank test, pairing scores sentence‐by‐sentence. AirPods performance in Spanish versus Mandarin was compared with a Mann–Whitney *U* test.

## 3 Results

A total of 116 Spanish and 91 Mandarin sentences were evaluated across three encounters. In Spanish, mean scores for AirPods were: fluency 4.94, adequacy 4.86, meaning 4.82, and severity 4.91 (Table 1). Means for Meta were 4.62, 4.65, 4.49, and 4.71, respectively.

**TABLE 1:** AirPods vs. Meta (Spanish, *n* = 116): Descriptive statistics and Wilcoxon signed‐rank test.

Metric	AirPods Mean (SD) [95% CI]	Meta Mean (SD) [95% CI]	Meta < AirPods	Meta > AirPods	Ties	*Z*	*p*
Fluency	4.94 (0.33) [4.87, 4.99]	4.62 (0.68) [4.48, 4.73]	35	3	78	−5.069	<0.001
Adequacy	4.86 (0.56) [4.76, 4.95]	4.65 (0.81) [4.50, 4.78]	20	6	90	−2.646	0.008
Meaning	4.82 (0.54) [4.72, 4.91]	4.49 (1.00) [4.30, 4.66]	27	9	80	−3.260	0.001
Severity	4.91 (0.40) [4.83, 4.97]	4.71 (0.75) [4.56, 4.84]	17	5	94	−2.859	0.004

*Note*: Based on positive ranks. Negative *Z* values indicate Meta scored lower than AirPods.

AirPods performed better in all four categories (*p* < 0.05) compared to Meta (Table 1) and performed better in Spanish than in Mandarin: fluency (*Z* = −5.069, *p* < 0.001), adequacy (*Z* = −2.646, *p* = 0.008), meaning (*Z* = −3.260, *p* < 0.001), and error severity (*Z* = −2.859, *p* = 0.004) (Table 2). In Spanish, AirPods made two clinically significant errors (severity 1–3) across all three encounters, while Meta made seven. In Mandarin, AirPods made nine significant errors across three encounters.

**TABLE 2:** AirPods Spanish vs. Mandarin: Descriptive statistics and Mann–Whitney *U* test.

Metric	Spanish (*n* = 116) Mean (SD) [95% CI]	Mandarin (*n* = 91) Mean (SD) [95% CI]	*U*	*Z*	*p*
Fluency	4.94 (0.33) [4.87, 4.99]	4.71 (0.70) [4.57, 4.85]	4514.5	−3.339	<0.001
Adequacy	4.86 (0.56) [4.76, 4.95]	4.47 (0.89) [4.29, 4.65]	3852.5	−4.800	<0.001
Meaning	4.82 (0.54) [4.72, 4.91]	4.32 (1.08) [4.09, 4.54]	4003.5	−4.100	<0.001
Severity	4.91 (0.40) [4.83, 4.97]	4.59 (0.84) [4.41, 4.76]	4350.5	−3.546	<0.001

*Note*: Negative *Z* values indicate Mandarin scored lower than Spanish.

Each encounter took, on average, 8.34 min in Spanish and 7.76 min in Mandarin with an in‐person translator. Encounters in Spanish took 5.62 min with AirPods, and 7.18 min with Meta, resulting in 2.72 min (32%) and 1.16 min (14%) saved, respectively. In Mandarin, encounters took 6.18 min with AirPods, resulting in 1.58 min saved (20%). In terms of usability, Meta scored 50.0 on the 100‐point SUS scale, and AirPods scored 92.5. AirPods scored above the standard threshold of 68 considered adequate for usability [6].

## 4 Discussion

Both devices scored highly but still made clinically significant errors. AirPods performed better in Spanish, consistent with the literature, potentially due to training data availability and syntactic similarity to English [4]. Common errors included the device “mishearing” words, such as translating “liver disease” to “liberty disease,” or “flow” to “throat.” Devices struggled with figures of speech, translating “your eyes may water” to “more water may flow out of your eye” in Mandarin and “blowing your nose” to “blowing with wind.” A clinically severe error included the device's failure to hear the word “not,” reversing an instruction. Both devices handled medical terminology well, accurately translating “Tylenol,” “Eliquis,” “chronic rhinosinusitis,” “nasal polyps,” “cauterize,” “nasal septum,” “decongestant,” and “endoscopy,” while struggling on a minority of terms such as “labetalol.”

Most literature suggests that interpreters require 5–10 extra minutes of visit time per 15–30+ minute visit, though a minority suggests no increased time [7, 8]. In our study, live‐translation saved 14%–32% of visit time compared to a human translator. In‐person translators range from $45 to $150/h, and phone services may cost $1.25–$3.00/min [9]. Costs of live‐translation technologies are not insignificant ($249 for AirPods Pro 3, $379 for Meta, and $1000+ for AI‐enabled smartphones), but these one‐time costs may eventually be worthwhile investments.

Meta glasses had a low usability score (50.0), which may be due to the less intuitive user interface and the lack of sound isolation inherent to headphones. Another usability barrier is that both technologies require two devices and two smartphones per patient encounter. Additionally, limited English proficiency (LEP) patients face barriers to health literacy that in‐person interpreters may be better able to accommodate [10].

This study was limited by interpreter availability, and four interpreters graded encounters interchangeably, contributing to scoring variability. Encounters were short and performed under ideal conditions. Next steps could include a technology company partnership with physicians to ensure HIPAA compliance and accurate term dictionaries, and with medical translators to ensure contextual accuracy. Caution is warranted against a hasty rollout of AI‐assisted translation technologies in the name of time or cost savings before these next steps are considered. Though there are practical barriers to the implementation of live translation, these technologies may transform access to medical care for LEP patients.

## Funding

The authors received no specific grant from any funding agency to sponsor this work.

## Ethics Statement

This prospective study was deemed exempt from institutional review board review at the Thomas Jefferson University Hospitals.

## Conflicts of Interest

Elina Toskala has received consulting fees from Aerin, a research grant from GSK, and was a member of the speaker's bureau for Sanofi. Gurston Nyquist has received consulting fees from Sanofi. Mindy Rabinowitz has been a consultant to Medtronic and Integra Life Sciences. The remaining authors have nothing to disclose.
